# Generalizability of clinical prediction models in mental health

**DOI:** 10.1038/s41380-025-02950-0

**Published:** 2025-03-19

**Authors:** Maike Richter, Daniel Emden, Ramona Leenings, Nils R. Winter, Rafael Mikolajczyk, Janka Massag, Esther Zwiky, Tiana Borgers, Ronny Redlich, Nikolaos Koutsouleris, Renata Falguera, Sharmili Edwin Thanarajah, Frank Padberg, Matthias A. Reinhard, Mitja D. Back, Nexhmedin Morina, Ulrike Buhlmann, Tilo Kircher, Udo Dannlowski, MBB consortium, Maike Richter, Daniel Emden, Ramona Leenings, Janette Ratzsch, Rogério Blitz, Lena Florentine Köhler, Moritz Rau, Nils Opel, FOR2107 consortium, Tilo Kircher, Udo Dannlowski, Katharina Thiel, Kira Flinkenflügel, Navid Schürmeyer, Anna Kraus, Janik Goltermann, Igor Nenadic, Benjamin Straube, Nina Alexander, Hamidreza Jamalabadi, Andreas Jansen, Frederike Stein, Florian Thomas-Odenthal, Paula Usemann, Lea Teutenberg, Katharina Brosch, Susanne Meinert, PRONIA consortium, Nikolaos Koutsouleris, Paolo Brambilla, Rachel Upthegrove, Franco Fabbro, Raimo K. R. Salonkangas, Joseph Kambeitz, Stefan Borgwardt, Eva Meisenzahl-Lechner, Alessandro Bertolino, Rebekka Lencer, Tim Hahn, Nils Opel

**Affiliations:** 1 Department of Psychiatry and Psychotherapy, Jena University Hospital, Jena, Germany; 2 Institute for Translational Psychiatry, University of Münster, Münster, Germany; 3 German Center for Mental Health (DZPG), Site Jena-Magdeburg-Halle, Germany; 4 Center for Intervention and Research on adaptive and maladaptive brain Circuits underlying mental health (C-I-R-C), Jena-Magdeburg-, Halle, Germany; 5 Institute of Medical Epidemiology, Biometrics, and Informatics, Interdisciplinary Center for Health Sciences, Medical School of the Martin Luther University Halle-Wittenberg, Halle (Saale), Germany; 6 Department of Psychology, Martin Luther University Halle-Wittenberg, Halle (Saale), Germany; 7 Department of Psychiatry and Psychotherapy, University Hospital LMU Munich, Munich, Germany; 8 German Center for Mental Health (DZPG), Site Munich-Augsburg, Germany; 9 Department of Psychosis Studies, Institute of Psychiatry, Psychology and Neuroscience, King’s College London, London, UK; 10 Max Planck Institute of Psychiatry, Munich, Germany; 11 Department for Psychiatry, Psychosomatic Medicine and Psychotherapy, University Hospital Frankfurt, Goethe University, Frankfurt am Main, Germany; 12 Max Planck Institute for Metabolism Research, Cologne, Germany; 13 Institute of Psychology, University of Münster, Münster, Germany; 14 Joint Institute for Individualisation in a Changing Environment (JICE), University of Münster and Bielefeld University, Site Münster-Bielefeld, Germany; 15 Department of Psychiatry, University of Marburg, Marburg, Germany; 16 Institute of Behavioral Sciences, Feinstein Institutes for Medical Research, Manhasset, NY USA; 17 Institute for Translational Neuroscience, University of Münster, Münster, Germany; 18 Department of Pathophysiology and Transplantation, University of Milan, Milan, Italy; 19 Institute for Mental Health and Centre for Human Brain Health, School of Psychology, University of Birmingham, Birmingham, UK; 20 Department of Psychiatry, University of Oxford, Oxford, UK; 21 Department of Psychiatry, University of Udine, Udine, Italy; 22 Department of Psychiatry, University of Turku, Turku, Finland; 23 Department of Psychiatry and Psychotherapy, Faculty of Medicine and University Hospital, University of Cologne, Cologne, Germany; 24 Department of Psychiatry, Psychiatric University Hospital, University of Basel, Basel, Switzerland; 25 Department of Psychiatry and Psychotherapy, Medical Faculty, Heinrich-Heine University, Düsseldorf, Germany; 26 Department of Basic Medical Science, Neuroscience, and Sense Organs, University of Bari Aldo Moro, Bari, Italy; 27 Department of Psychiatry and Psychotherapy, University of Münster, Münster, Germany

**Keywords:** Depression, Diagnostic markers

## Abstract

Concerns about the generalizability of machine learning models in mental health arise, partly due to sampling effects and data disparities between research cohorts and real-world populations. We aimed to investigate whether a machine learning model trained solely on easily accessible and low-cost clinical data can predict depressive symptom severity in unseen, independent datasets from various research and real-world clinical contexts. This observational multi-cohort study included 3021 participants (62.03% females, *M*_Age_ = 36.27 years, range 15–81) from ten European research and clinical settings, all diagnosed with an affective disorder. We firstly compared research and real-world inpatients from the same treatment center using 76 clinical and sociodemographic variables. An elastic net algorithm with ten-fold cross-validation was then applied to develop a sparse machine learning model for predicting depression severity based on the top five features (global functioning, extraversion, neuroticism, emotional abuse in childhood, and somatization). Model generalizability was tested across nine external samples. The model reliably predicted depression severity across all samples (*r* = 0.60, *SD* = 0.089, *p* < 0.0001) and in each individual external sample, ranging in performance from *r* = 0.48 in a real-world general population sample to *r* = 0.73 in real-world inpatients. These results suggest that machine learning models trained on sparse clinical data have the potential to predict illness severity across diverse settings, offering insights that could inform the development of more generalizable tools for use in routine psychiatric data analysis.

## Introduction

The inability to predict the occurrence of depressive symptoms and patients’ individual trajectories remains a major limitation in mental health care. Generating data-driven support for clinical decision-making is therefore the main objective of many recent advances in psychiatric research [1]. To achieve this goal, we require machine learning (ML) models that are able to identify consistent patterns in predictors of depression severity from the complex inter-individual variety found in real-world clinical populations. A particular challenge for the field is the development of models that not only make reliable predictions within the participant cohort used for model training, but that are also valid in unseen, independent data from different treatment contexts, countries, or age groups [2, 3]. While models for clinically relevant predictions have been successfully trained within a single research dataset [4–6], previous investigations have often overlooked external validation, specifically in real-world samples, which represent the populations for whom clinical models are developed and should be applicable [7]. Recently, attempts at externally validating models for treatment response prediction have failed, raising concerns about their generalizability [8, 9].

A potential pitfall may lie in systematic differences between data from real-world clinical populations and those derived from research cohorts, as clinical and demographic sample differences can impair prediction accuracy and model generalizability [3, 10–12]. Although imaging and genetic data have proven invaluable for advancing precision medicine outside of mental health [13–16], previous psychiatric research has repeatedly demonstrated the particular relevance of training models on clinical data when predicting symptom trajectories and treatment outcome in disorders such as schizophrenia or depression [17, 18]. However, despite the technical feasibility of implementing structured collection of clinical information, the widespread absence of harmonized machine-readable clinical data persists across research and treatment settings, primarily due to a lack of uniform data standards and shared ontologies in psychiatry [19].

Given that the generalizability of ML models for clinical applications like predicting treatment response has recently been questioned [8], it is crucial to first assess whether robust and generalizable models can be developed to predict depressive symptom severity across diverse samples. If sampling biases or batch effects impede model generalizability to the extent that generalizable cross-sectional symptom prediction using clinical data is not possible, then a re-evaluation of our current direction is imperative. We therefore need to improve our understanding of the differences between study populations and real-world data and investigate the generalizability of predictive models for depressive symptoms in unseen, independent data from various sites and settings as a foundation, before taking on the even more complex challenges of predicting symptom trajectories in response to intervention.

In this study, we investigate whether a ML model for the cross-sectional prediction of depressive symptoms, trained on structured clinical information, can achieve generalization across diverse samples, sites, and time points despite potential sampling and treatment effects. Specifically, we aimed to train a ML model on homogenous research data and systematically validate it on independent research and real-world clinical data obtained from both inpatient and outpatient settings, as well as from the general population.

## Materials and methods

### Study design and participants

This was a cross-sectional multi-center study in ten independent samples with an overall n = 3021. From May 2010 to February 2024, 3021 participants aged 15–81 were included as part of ten different studies or real-world data collection efforts. All inpatient and outpatient participants were diagnosed with major depressive disorder (MDD). Participants across all illness stages were included, ranging from one sample comprising participants with recent onset depression (ROD, n = 301), eight samples including a more general range of MDD diagnoses (range of n: 43-1210), to one sample with persistent depressive disorder (PDD, n = 161). All participants were undergoing inpatient or outpatient treatment at the time of assessment, with the exception of the real-world general population sample, from which participants were selected who reported having received an MDD diagnosis at some point before the assessment. An overview of all samples including descriptive and clinical information can be found in Table 1. We firstly evaluated sampling effects across patients from two base samples: a study population and a real-world sample recruited at the same psychiatric hospital to eliminate site variability: For the study sample (study population inpatients, site #1), we used clinical and self-report data from two pooled neuroimaging cohorts conducted at the same site with virtually identical data assessment protocols. For comparison, a sample from a naturalistic study of a real-world clinical population that was digitally phenotyped during inpatient treatment at the same psychiatric hospital was included (real-world inpatients, site #1). We then included seven additional samples from various sites across Germany and one sample containing data from multiple sites across Europe, deviating further from the study population in terms of patient characteristics and recruitment setting with each site (see Supplementary Material (SM), pp. 5–8). All samples are findable through the Meta-Data Study Repository of the German Centre for Mental Health (DZPG) (http://www.umh.de/cohort-registry). The study protocol was approved by the responsible ethics committees and was conducted in accordance with the guidelines for good clinical practice.

**Table 1:** Overview and descriptive information for all sites.

Sample	Recruitment site	Diagnosis	Intervention	Treatment Duration in Days	Age	Sex	Baseline Depression	Depression FU	Extra-version	Neuro-ticism	GAF	Somati-zation	CTQ EA
				Mean (SD)	Range Mean (SD)	m/f	Mean (SD)	Mean (SD)	Mean (SD)	Mean (SD)	Mean (SD)	Mean (SD)	Mean (SD)
Study Population Samples													
Study population inpatients, site #1 (n = 366)	Department of Psychiatry, University Hospital Münster, Germany	Acute MDD, all severity categories	Medication,CBT	NA	18–65 37.16 (12.86)	158/208	39.82 (17.02)	NA	39.08 (14.53)	66.95 (15.50)	54.43 (9.37)	11.69 (7.79)	11.18 (5.50)
Study population in-& outpatients, site #1 (n = 83)	Department of Psychiatry, University Hospital Münster, Germany	Acute MDD, all severity categories	Medication,ECT, CBT	167.24 (108.71)	19–66 33.72 (12.13)	28/55	34.17 (18.81)	26.29 (16.92)	NA	NA	57.84 (12.63)	NA	11.19 (5.57)
Study population inpatients, site #2 (n = 109)	Department of Psychiatry, University of Marburg, Germany	Acute MDD, all severity categories	Medication,CBT	NA	18–63 36.75 (13.18)	50/59	36.43 (16.39)	NA	45.24 (16.13)	66.03 (15.36)	54.96 (8.89)	14.05 (8.69)	11.41 (5.25)
Study population inpatients, site #3 (n = 43)	Department of Psychiatry and Psychotherapy, Jena University Hospital, Germany	Acute MDD, all severity categories	Medication,CBT	NA	18–67 39.23 (15.58)	18/24	49.65 (20.07)	NA	32.71 (10.58)	56.04 (14.78)	42.85 (11.35)	NA	11.62 (5.74)
Study population in- & outpatients, multisite (n = 301)	Ten international recruitment sites^a^	Recent onset depression	Medication, psychotherapy, counseling	NA	15–41 25.4 (6.11)	155/146	39.88 (19.37)	NA	47.12 (16.19)	64.14 (17.33)	54.02 (12.28)	NA	9.21 (4.22)
Real-World Samples													
Real-world inpatients, site #1 (n = 352)	Department of Psychiatry, University Hospital Münster, Germany	Acute MDD, all severity categories	Medication,CBT	44.67 (23.23)	18–81 39.3 (17.22)	165/187	39.40 (18.05)	21.38 (18.57)	36.17 (18.78)	68.60 (17.32)	53.86 (9.18)	12.22 (7.75)	11.51 (5.79)
Real-world inpatients, site #4 (n = 161)	Department of Psychiatry and Psychotherapy, Ludwig-Maximilian University Munich, Germany	Persistent depressive disorder	Medication,10-week CBASP	70.98 (8.12)	18–66 39.33 (12.55)	68/93	48.95 (16.77)	36.10 (21.41)	33.81 (14.51)	71.26 (13.77)	46.42 (8.25)	NA	14.40 (6.01)
Real-world outpatients, site #5 (n = 144)	Psychotherapeutic Outpatient Unit, Martin Luther University Halle-Wittenberg, Germany	Acute MDD, all severity categories	Medication,CBT	191.98 (12.25)	19–60 28.76 (9.72)	32/112	32.47 (17.32)	19.07 (17.56)	NA	NA	62.11 (11.68)	8.07 (6.09)	10.72 (4.76)
Real-world outpatients, site #6 (n = 252)	Psychotherapeutic Outpatient Unit, University of Münster, Germany	Acute MDD, all severity categories	Medication,CBT	613.91 (338.53)	19–64 31.99 (11.38)	105/147	37.84 (16.39)	15.16 (14.35)	NA	NA	NA	9.47 (7.56)	NA
Real-world general population sample (n = 1210)	Institute of Medical Epidemiology, Medical Faculty of the Martin Luther University Halle-Wittenberg, Germany	Self-reported MDD diagnosis in lifetime	NA	NA	20–72 51.10 (11.08)	367/843	25.63 (19.23)	NA	50.42 (19.66)	48.56 (19.13)	NA	NA	NA

*CBASP* cognitive behavioral analysis system of psychotherapy, *CTQ EA* childhood trauma questionnaire, emotional abuse subscale, *ECT* electro-convulsive therapy, *GAF* global assessment of functioning, *Depression* percentage of maximum possible depression severity score, calculated from beck depression inventory or patient health questionnaire, depression scale, *Depression FU* depression severity after treatment, *Extraversion* percentage of maximum possible extraversion score, calculated from big five inventory-S, big five inventory-2-S, NEO-five factor inventory, *MDD* major depressive disorder, *Somatization* symptom checklist 90-revised, somatization subscale.

^a^see Supplementary Material for details.

### Procedures

To capture heterogeneity and diversity of real-world patient populations, samples included participants with persistent depressive disorder (PDD) undergoing specialized treatment with cognitive behavioral analysis system of psychotherapy (CBASP), inpatient samples undergoing electroconvulsive therapy (ECT), inpatient and outpatient participants with recent onset depression (ROD) and outpatient samples from psychotherapy services undergoing long-term psychotherapeutic treatment. More details on all treatment procedures can be found in the SM, pp. 5–8.

### Measures

All available data were extracted and retained as predictor variables for the training of ML models if they were available in both initial samples. This resulted in a set of 76 variables that could be grouped into the following dimensions: sociodemographic variables, current symptom severity, current psychotropic medication, family and personal psychiatric history, childhood maltreatment and stressful life events, somatic symptoms, and personality dimensions. Symptomatic outcomes were assessed based on harmonized scores from self-report measures of depression severity for all sites. As depression severity was assessed with different instruments (BDI [20], BDI-II [21], or PHQ-9 [22]) across different sites, we harmonized these measures by transforming them into absolute percent of maximum possible (POMP) scores. The score represents the percentage a participant achieved in relation to the maximum possible depression severity that can be achieved in the measurement tool [23] (see SM, pp. 4–5). Where available, depression severity after a psychotherapeutic intervention or at the conclusion of treatment was additionally included for model validation across time-points. Detailed descriptions of all measures are presented in SM, pp. 3–5.

### Statistical analysis

As a first analysis step, we calculated group comparisons between the two base samples: study population and real-world inpatients from site #1. Independent two-sample t-tests were calculated for continuous, *Χ*²-tests for dichotomous, and Mann-Whitney-U test for ordinal outcome variables. Benjamini-Hochberg false discovery rate (FDR)-corrected p-values were calculated for all comparisons. Statistics were computed using IBM SPSS Version 26.

**Fig. 1:**
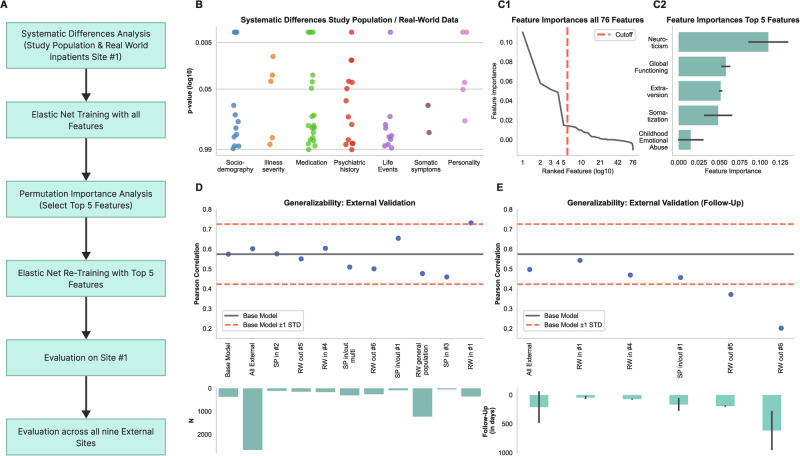
Analytic workflow, model evaluation, and results of multisite model validation. **A** Analytic workflow from systematic differences analysis to multisite model evaluation. **B** Scatter plot depicting p-values for group differences between study population and real-world inpatients from site #1 across clinical and demographic variables. **C1** Line plot of ranked feature importances with specified cutoff. **C2** Bar plot highlighting the top 5 features selected through permutation importance analysis. **D** External validation results of the base model showing Pearson correlation of true and predicted depressive symptoms, contrasted across nine external sites. **E** Follow-up validation scatter plot showing Pearson correlation of true and predicted depressive symptoms following therapeutic intervention, including the presentation of average follow-up durations by site.

For the ML analysis, we first trained a model on all N = 366 study population inpatients #1, using all available 76 features to predict depression severity. Analogous to Chekroud et al. [8], we used the elastic net algorithm, a penalized regression method that is appropriate when covariates are correlated with one another and predictors may only be sparsely endorsed [24, 25]. We performed cross-validation to assess generalizability of our model using the PHOTONAI software (www.photon-ai.com [26]). The cross-validation part of this procedure randomly reshuffled the data, separated the dataset into 10 non-overlapping folds and used 9 of the subsets for training, repeating the process until each subset was left out once for testing. The repeated part of this procedure randomly re-shuffled and re-split the data ten times to reduce the impact of the first random data split; in aggregate, 100 total models were fit to the 10 folds by 10 repeats. Missing values were imputed using the median of the training set within the cross-validation procedure, preserving the independence of training and test set. Model performance was calculated by averaging the performance metrics across all 100 models. Based on the prediction of this baseline model, we computed Pearson correlations between the true and the predicted values to assess predictive performance. Next, we identified the most relevant features for this model using permutation importance with 1000 repeats. This yielded five main variables driving model performance (Fig. 1): neuroticism, extraversion, global assessment of functioning, somatization, and emotional abuse during childhood. Using these five variables alone, we trained a sparse model on the same initial sample (study population inpatients #1). We then tested this sparse model in real-world inpatients #1. If missing values were present, the median of the base model dataset was used. To further assess model generalizability, we then tested the sparse model across all nine external samples for external validation (see Fig. 1). To assess whether model performance remained robust after therapeutic interventions, we used it to predict depression severity after treatment across the five external datasets which provided an assessment after a therapeutic intervention (see Table 1). For all models, we computed the Binomial Effect Size Display (BESD [27]) from the Pearson correlation coefficients. This metric provides an approximation of the proportion of correct guesses about the direction of a correlation. It adjusts the initial chance level (50%) by incorporating the strength of the correlation coefficient, as a metric for evaluating the coefficient’s practical significance. More specifically, BESD is a tool to make the interpretation of correlation coefficients (r) more intuitive, especially when dealing with binary outcomes (e.g., success/failure). It converts a correlation into success rates for two groups - those predicted to succeed and those predicted to fail. Conversion is done in two steps:

1. Baseline (no correlation, r = 0): Assume a 50% success rate for both groups.
2. Adjust for r: For a given correlation:
  Success rate for the “success-predicted” group = (50 + 50r)%
  Success rate for the “failure-predicted” group = (50−50r)%

Random chance occurs when the correlation r = 0. This means there is no relationship between the predictor and the outcome. Under this condition, both groups (those predicted to succeed and those predicted to fail) have the same success rate of 50%. This reflects pure random guessing.

So, if r = 0:

- Success rate in the success-predicted group = 50 + (50*0) = 50%
- Success rate in the failure-predicted group = 50−(50*0) = 50%

But if, for example, r = 0.20:

- Success rate in the success-predicted group = 50 + (50 * 0.20) = 60%
- Success rate in the failure-predicted group = (50 − (50 * 0.20) = 40%

Thus, r = 0.20 means a 20% difference in success rates, making the effect size easier to interpret.

## Results

### Systematic comparison between study population and real-world sample

The two base samples differed substantially in features from all dimensions except for somatic symptoms. The real-world participants displayed more severe current depressive symptoms only in external symptom assessment, not in a self-report measure. They also showed a more severe disease course, as well as differences in prescribed medication (more stimulants, benzodiazepines, and z-drugs), recalled childhood maltreatment (more physical neglect) and personality dimensions (lower conscientiousness, higher agreeableness) compared to the study population (see SM, Table S2 for all group comparisons). In addition to this comparison between the two initial samples from the same treatment site, we also compared all external validation samples regarding their deviation from the training sample in the top five features. These results are reported in the SM, p. 9.

### Real-World validation of ML model and development of sparse model

Training the first ML model on all available data in study population inpatients #1 yielded an internal validation performance of Pearson’s *r*_364_ = 0.57, *SD* = 0.151. As outlined above, the five most important features were then used to train the sparse model on study population inpatients #1. The sparse model performed above chance in the real-world sample (*r*_350_ = 0.73, *p* < 0.001). Using the BESD for illustration, this corresponds to an accuracy of 87% in a classification scenario.

### Generalizability of sparse model across sites, treatment settings, and populations

The sparse model also performed above chance level across all external datasets (*r*_2673_ = 0.60, *SD* = 0.089, *p* < 0.001), corresponding to an accuracy of 80% in a classification scenario. Investigating performance on the nine samples separately shows that performance on all sites varied between *r*_1227_ = 0.48 in the real-world general population sample, *r*_250_ = 0.50 in real-world outpatients #6, and *r*_350_ = 0.73 in real-world inpatients #1. Thus, even the lowest performance was within 0.60 standard deviations of the mean of the first model performance. Note that the comparatively poorer performance in the real-world general population sample may result from only two of the five features being available for this sample, which moreover differed most markedly from the training set in participant characteristics due to it being a general population sample in which participants were not necessarily acutely depressed or currently undergoing treatment.

### Model generalizability across two time points

To assess whether sparse model performance remained robust after therapeutic interventions, we used it to predict depression severity after treatment. The sparse model performed above chance level (*r*_566_ = 0.50, *p* < 0.001) across the five external datasets which provide an assessment after a therapeutic intervention, which corresponds to an accuracy of 75% in a classification scenario and indicates good generalization for the prediction of depression severity at a different measurement time without explicit training. Investigating performance on the five sites separately showed that performance varied between *r*_125_ = 0.20 (real-world outpatients #6) and *r*_56_ = 0.54 (real-world inpatients #1). Note that treatment duration differed substantially between sites and treatment modalities. The comparatively low performance in real-world outpatients #6 may be due to the long treatment as treatment duration was indeed positively associated with model error across all sites, indicating increased model error with longer duration between baseline and follow-up assessment (Spearman’s *r*_554_ = 0.12, *p* = 0.004).

### Classification of severely depressed non-responders

As the predictive model showed robust performance for depressive symptom prediction at two distinct time points before and after intervention, we additionally aimed to assess whether the same variables could be used to train a model to identify subjects with severe depressive symptoms at both time points thus allowing to assess its potential value for individual risk assessment. We used the established BDI cut-off of 29, indicating severe depression [21], which corresponded to a POMP score of 46.03 to stratify the sample of 790 patients for whom data from two time-points was available, yielding 91 (13%) who showed severe depressive symptoms at both time-points. While training a baseline model on the study population inpatients #1 dataset alone and testing its generalization to the nine other sites was not feasible as study population inpatients #1 contained only 19 patients who were severely depressed at both time-points, we assessed our ability to predict severe depression without treatment response using leave-site-out cross-validation. In this procedure, data from all but one site is used for training and the model is tested on the remaining site. This is repeated for each site. To counter the strong class imbalance, we employed the elastic net approach for classification with Synthetic Minority Over-sampling Technique (SMOTE) combined with Edited Nearest Neighbors (ENN) as proposed by Batista et. al [28]. With this approach, we showed that presence/absence of persistent, severe depression could be predicted with an average balanced accuracy of 0.66. Performance per site ranged from chance level (balanced accuracy = 0.50) in real-world outpatients #6 to 0.86 in study population inpatients #2 (SM p. 20, Table S3).

### Sensitivity analyses

To investigate potential model bias, we assessed the association of model error with age and sex, respectively. Neither age (Spearman’s *r*_554_ = 0.07, *p* = 0.093) nor sex (*t*_554_ = −1.54, *p* = 0.123) were significantly associated with model error. We also conducted an additional analysis to explicitly test the generalization performance of our model when excluding neuroticism and global functioning, indicating good generalization even without the most highly weighted features of the original model (see SM, p. 9)

## Discussion

In this study, we demonstrate that a ML model trained on homogeneous research data can achieve comparable performance for predicting depression severity in unseen, independent real-world datasets across different sites, treatment settings, and time points. To the best of our knowledge, this study includes the most extensive independent validation of a ML model in the field of psychiatric research to date. In contrast to previous studies [8, 17], we show robust generalization performance across nine independent sites comprising over 2600 participants, reflecting the full spectrum of heterogeneity and diversity present in real-world patient populations. This suggests that real-world validation of psychiatric symptom prediction models is possible, despite substantial sample heterogeneity.

A first challenge to consider for model generalization in independent datasets is that patient groups from research contexts may be too different from real-world clinical populations [29]. We demonstrate that systematic differences indeed exist between research populations and real-world MDD patients, even when both samples are treated and assessed at the same psychiatric hospital. However, our results suggest that these differences do not impede model generalization, even to populations from different sites or treatment contexts. While research from other areas of medicine, such as predicting positive COVID-19 screenings, reveals that site-specific model customization can improve predictive performance, the approach of applying a ready-made model “as-is” has also been found to be effective [30] and appears to also be feasible for psychiatry. It should be noted however that most of the real-world data used in this study were derived from naturalistic scientific investigations and therefore still relied on patients’ voluntary participation. This may have introduced inherent biases, as more strongly impaired subgroups, such as patients in closed wards or those with suicidal tendencies, were inevitably excluded. Sample characteristics of the whole clinical population may therefore deviate more strongly from study data and face more difficulties for generalization than we are able to report.

Biases arise not only from baseline differences in patient characteristics and site but also from variations in treatment modalities, especially for prospective predictions of depression severity after a mental health intervention. We show that our model remains robust across various settings, particularly for the translation from inpatient to outpatient psychotherapy service users, as well as after treatment with markedly different modalities. While performance drops markedly the further the treatment context deviates from the training set and with increasing time between baseline and follow-up assessment, prediction of both baseline as well as post-treatment depression severity is still possible. This underlines the finding that heterogeneity within and between datasets and measurement time does not substantially impede model generalizability. However, although the predictive clinical features used in our sparse model may allow for the identification of participants with persistent depressive symptoms across time points and after treatment — and similar approaches have been used to tailor treatments for participants with more intensive support needs during routine treatment [31] — the limited sample size of treatment resistant participants did not allow for external validation in this additional analysis. This model should therefore not be misinterpreted as a readily applicable model for clinical decision support. The present findings rather support the general feasibility of developing generalizable ML models for predicting complex phenomena such as psychiatric symptoms. These findings may thus serve as a foundational step for future endeavors aimed at refining models suitable for ecologically valid clinical use cases in daily practice.

A further challenge to consider is the quality, quantity, and diversity of the data needed to achieve accurate predictions. While previous research in study populations shows that predictive models which include more than one data modality, such as clinical, neuroimaging, and genetic data, achieve better performance [32] we demonstrate that symptom severity prediction is possible with sparse features that can be collected during the clinical routine. This is in line with previous findings on the particular importance of clinical information when predicting symptom trajectories and treatment outcome in mental health research [17, 18]. The extracted features, encompassing two personality dimensions, somatic symptom severity, childhood emotional abuse, and global functioning, and thus a mixture of state and trait variables, consistently form a predictive pattern for depression severity across diverse patient populations, irrespective of illness stage or treatment setting. It is crucial to highlight that these features have demonstrated greater importance compared to over 70 other variables, some of which might be presumed to hold equal or greater relevance in determining depressive symptom severity including clinician-relevant factors like psychiatric history or prescribed medication. However, note that the initial feature selection may not encompass the full spectrum of variables with predictive potential and that there may be other variables of greater significance that were not measured and therefore not included in the model.

Lastly, in addition to considerations about sample heterogeneity, a crucial methodological challenge for constructing generalizable ML models lies in the avoidance of overfitting when training the base model [29]. When a model overfits, it captures both the signal and the noise in the training data on which it may perform exceptionally well while failing to generalize to new, unseen data [33]. Regularization, which imposes constraints on the model parameters to encourage sparsity, can help prevent overfitting by promoting simpler, more interpretable models. In our study, working with low-dimensional clinical data and further reducing the dimensionality of the feature space by focusing on the most informative features was used to prevent overfitting.

Given our demonstration of the generalizability of ML models trained on sparse clinical information, along with considerations of technical and cost efficiency, these findings should advocate for the structured acquisition of machine-readable clinical information in routine settings. To achieve this, it is essential to enhance interoperability and invest in standardized data formats and ontologies in psychiatry, paving the way for the application of ML models across diverse clinical sites. Successful examples from the medical community include the adoption of the Systematized Nomenclature of Medicine, Clinical Terms (SNOMED CT [34]), Logical Observation Identifiers, Names, and Codes (LOINC [35]), and Fast Health Interoperability Resources (FHIR [36]) profiles. Moreover, wide-reaching infrastructures such as the German Medical Informatics Initiative [37] as well as other international efforts [38–40] have set the goal of improving clinical data integration from patient care and medical research. The French Health Data Hub, for instance, is explicitly designed to facilitate health data sharing with the aim of developing health-related Artificial Intelligence projects [41]. Our findings highlight the necessity for national and international initiatives to specifically tailor, develop, and disseminate such solutions for psychiatry and mental health. The recent establishment of the German Centre for Mental Health (DZPG) with its translational agenda and integration with key data infrastructures in Germany signifies an important step forward in this regard [42].

In summary, our findings highlight successful real-world validation of a sparse ML model for depressive symptom prediction and emphasize the potential of using standardized collection of routine data to develop generalizable empirical models in mental health.

## Supplementary information

Supplementary Material

## Data Availability

All samples and their data are findable and requestable through the Meta-Data Study Repository of the German Centre for Mental Health (DZPG) (http://www.umh.de/cohort-registry). The machine learning model is published in the PHOTONAI model repository (https://photon-ai.com/model_repo/generalizability-model). The corresponding author can be contacted for further information.

## Code availability

The model can be found in the PHOTONAI repository (https://photon-ai.com/model_repo/generalizability-model).

## Study approval

The research in all samples was conducted ethically in accordance with the World Medical Association Declaration of Helsinki and approved by all samples’ local institutional review boards and ethics committees named hereafter: • Ethikkommission beider Basel (EKBB), • Klinisches Ethikkomitee der Universitären Psychiatrischen Kliniken Basel (KLINEK UPK), • Ethics Commission of the Westphalia-Lippe Medical Association and the University of Münster, • Ethics Committee of the Faculty of Psychology and Sports Science at the University of Münster, • Ethics Commission of the Faculty of Medicine at the University of Münster, • Ethics Commission of the Faculty of Medicine at the Philipps-University Marburg, • Ethics Commission of the Faculty of Medicine of Friedrich-Schiller-University Jena, • Ethics Commission of the Faculty of Medicine at Ludwig-Maximilians-University Munich, • Ethics Commission of the Faculty of Medicine at Martin-Luther-University Halle-Wittenberg, • Kommission für ethische Fragen der Wissenschaft der Martin-Luther-Universität Halle-Wittenberg (KeFW), • Ethics Commission of the Faculty of Medicine of Cologne University, • Ethics Committee of the Medical Faculty at Heinrich Heine University of Düsseldorf, • Ethics Commission of the University of Turku, • Independent Ethical Committee of the University of Bari Aldo Moro (Il Comitato Etico), • Departmental Commission for the Experimentation and Protection of the Person of the Department of Medicine of the University of Udine (IRB-DMED), • Ethics Committee of the University of Milan, • Ethics Committee of the University of Birmingham.
