# Supplementary material for: Generalizability of clinical prediction models in mental health
Source: Mol Psychiatry. 2025 Mar 19;30(8):3632–9. doi: 10.1038/s41380-025-02950-0 (PMC12240828; doi:10.1038/s41380-025-02950-0)
Supplement: Supplementary file 1 — Supplementary Material [file 41380_2025_2950_MOESM1_ESM.docx]

Supplementary Materials for

**Generalizability of Clinical Prediction Models in Mental Health**

Maike Richter†, Daniel Emden†, Ramona Leenings, Nils R. Winter, Rafael Mikolajczyk, Janka Massag, Esther Zwiky, Tiana Borgers, Ronny Redlich, Nikolaos Koutsouleris, Renata Falguera, Sharmili Edwin Thanarajah, Frank Padberg, Matthias A. Reinhard, Mitja D. Back, Nexhmedin Morina, Ulrike Buhlmann, Tilo Kircher, Udo Dannlowski, PRONIA consortium, FOR2107 consortium, MBB consortium, Tim Hahn^#^ & Nils Opel^#^

Corresponding author: maike.richter@med.uni-jena.de

**Content:**

Consortia member authors

Materials and Methods
Figs. S1 – S3
Tables S1 – S3

## Consortium member authors

MBB consortium

Maike Richter^1,2^, Daniel Emden^1,2^, Nils Opel^1,2,3,4^, Ramona Leenings^1,2^, Janette Ratzsch^1^, Rogério Blitz^1^, Lena Florentine Köhler^1^, Moritz Rau^1^

FOR2107 consortium

Udo Dannlowski^2,6^, Katharina Thiel^2^, Kira Flinkenflügel^2^, Navid Schürmeyer^2^, Anna Kraus^2^, Janik Goltermann^2^, Tilo Kircher^11^, Igor Nenadic^11^, Benjamin Straube^11^, Nina Alexander^11^, Hamidreza Jamalabadi^11^, Andreas Jansen^11^, Frederike Stein^11^, Florian Thomas-Odenthal^11^, Paula Usemann^11^, Lea Teutenberg^11^, Katharina Brosch^11,16^, Susanne Meinert^2,17^

^16^Institute of Behavioral Sciences, Feinstein Institutes for Medical Research, Manhasset, NY, USA

^17^Institute for Translational Neuroscience, University of Münster, Germany

PRONIA consortium

Nikolaos Koutsouleris^9,13,14,15^, Paolo Brambilla^18^, Rachel Upthegrove^19,20^, Franco Fabbro^21^, Raimo K. R. Salonkangas^22^, Joseph Kambeitz^23^, Stefan Borgwardt^24^, Eva Meisenzahl-Lechner^25^, Alessandro Bertolino^26^, Rebekka Lencer^27^

^18^Department of Pathophysiology and Transplantation, University of Milan, Italy

^19^Institute for Mental Health and Centre for Human Brain Health, School of Psychology, University of Birmingham, UK

^20^Department of Psychiatry, University of Oxford, UK

^21^Department of Psychiatry, University of Udine, Italy

^22^Department of Psychiatry, University of Turku, Finland

^23^Department of Psychiatry and Psychotherapy, Faculty of Medicine and University Hospital, University of Cologne, Germany

^24^Department of Psychiatry, Psychiatric University Hospital, University of Basel, Switzerland

^25^Department of Psychiatry and Psychotherapy, Medical Faculty, Heinrich-Heine University, Düsseldorf, Germany

^26^Department of Basic Medical Science, Neuroscience, and Sense Organs, University of Bari Aldo Moro, Italy

^27^Department of Psychiatry and Psychotherapy, University of Münster, Germany

# Methods

## Materials – Analysis Step 1

For the group comparison between study population and real-world inpatients from site #1, available data were grouped along the following eight dimensions:

***Sociodemographic variables***

In addition to age and self-reported gender, sociodemographic variables encompassed the number of children and siblings, the highest education degree of the participants and both their parents and the average monthly household income. It was recorded whether participants or their parents immigrated to Germany but no further race and ethnicity data were collected. The population size of the main place of residence during childhood and adolescence was also recorded. From the population size, an urbanicity score was calculated in which a score of 1 was assigned to rural areas with a population size below 10000, 2 – towns with a population size between 10000 and 100000, and 3 – cities with a population of over 100000 ^1^.

***Depressive symptom severity and level of functioning***

The current severity of depressive symptoms was assessed with Beck Depression Inventory (BDI) ^2^) as a self-report questionnaire with 21 items. The 17-item Hamilton Depression Scale (HAMD) ^3^ was carried out by trained raters as an objective measure of depression severity. Within this dimension, percentage of diagnosed single depressive episodes vs recurring depression and percentage of participants with psychiatric comorbidities were also investigated. Global Assessment of Functioning (GAF) ^4^ was used by trained raters in both samples to assess participants’ psychological, social, and occupational functioning on a scale from 1 (severely impaired) to 100 (extremely high functioning).

***Current psychotropic medication***

Type and dose of psychopharmacological treatment were recorded by the research team in study population inpatients and extracted from the electronic health record (EHR) in real-world inpatients #1. A strategy described by Redlich et al. ^5^ was subsequently applied: Each psychotropic medication was coded as absent (0), low (1; defined as equal or lower average dose), or high (2; defined as greater than average dose), relative to the midpoint of the daily dose range recommended by the Prescribers’ Digital Reference ^6^. A composite measure of total medication load was calculated, reflecting dose and variety of different medications taken, by summing all individual medication scores. Moreover, each drug was assigned to its medication class and the prescription rates of each medication class between the samples were compared.

***Family and own psychiatric history***

Patients provided a detailed psychiatric history, including number and duration of previous inpatient treatments, their age at the time they were first treated due to psychiatric symptoms and the duration they had spent on sick leave or in retirement due to mental illness. In study population inpatients, the main diagnosis as well as psychiatric comorbidities were verified via SCID-I performed by trained raters, whereas in real-world inpatients, all diagnoses were directly entered into the EHR by the treating physician after admission and initial clinical assessment.

To assess family history and genetic risk for mental disorders, patients were asked whether an immediate family member had been formally diagnosed with a mental illness and to choose the disorder from a list including MDD, bipolar disorder (BD), substance use disorders, anxiety disorders, post-traumatic stress disorder (PTSD), obsessive-compulsive disorder (OCD), psychotic disorders, attention-deficit (and hyperactivity) disorders (ADHD), eating disorders, or other.

***Childhood maltreatment and stressful life events***

The Childhood Trauma Questionnaire (CTQ) ^7^ was used as a retrospective self-report measure for childhood maltreatment. It contains 28 items that are scored on a five-point Likert scale. The five subscales of childhood maltreatment that are measured by this instrument are physical neglect, emotional neglect, and physical, emotional, and sexual abuse. As a measure of more recent stressors, the Life Events Questionnaire ^8^ was used in study population inpatients to assess whether participants had encountered stressful events (a separation or divorce, death of a loved one, losing their job, and moving house) in the last six months before participation and the impact (from – 3, “very negative” to + 3, “very positive”) this had on respondents. In real-world inpatients, a modified version of the questionnaire was used for brevity, in which the impact was not assessed. The groups were therefore only compared on the frequency of encountering stressful life events and not on their impact.

***Somatic symptoms***

Somatic symptoms in the last 4 weeks before study inclusion were assessed for both samples based on the somatization subscale of the Symptom Check List (SCL-90-R) ^9^ which consists of 12 items describing somatic complaints. Participants rated each item on a 5-point Likert scale (0, “no problem” to 4, “very serious”). Body mass index (BMI) was calculated from height and weight which study population inpatients self-reported whereas, in real-world inpatients, these measures were extracted from the EHR for those participants for whom they were available from clinical routine documentation. For real-world inpatients, somatic comorbidities as diagnosed by the treating physician were extracted from the EHR and participants also indicated their somatic ailments on a list of somatic disorder categories. In study population inpatients, an extensive list of somatic disorders was applied as exclusion criteria before initial participation. This information can be found in table S1.

***Big Five Personality Dimensions***

Big Five personality dimensions were assessed with self-report questionnaires. In study population inpatients, the 60-item NEO-FFI ^10^ was used while real-world inpatients completed the 30-item BFI-2-S ^11^. Although these questionnaires were both constructed to measure the same underlying constructs ^12^, they are not directly comparable due to diverging scale ranges. To achieve comparability, a procedure described by Fleeson & Gallagher (2009) ^13^ was used to transform NEO-FFI and BFI-2-S values into absolute percent of maximum possible (POMP) scores ^14^. The ‘pomp’ function from the ‘fancyr’ package in R was used for score transformation ^15^. The samples were subsequently compared along their Big Five dimensions using these scores.

## Materials – Analysis Step 2

All variables described above (with the exception of HAMD, which also measures depression severity) were included to train the first model in study population inpatients #1. This yielded five main features driving model performance. Using these five features alone, we trained the base model on all study population inpatients #1. We tested the base model based on the five features at external samples from different research and clinical settings and geographical sites. To make the variables comparable across different sites, some harmonization was conducted as outlined below:

***Outcome***

As depression severity, which was our outcome of interest, was assessed with different instruments (BDI ^2^, BDI-II ^16^, or PHQ-9 ^17^) across different sites, we harmonized these measures by transforming them into absolute percent of maximum possible (POMP) scores as described above. The score represents the percentage a participant achieved in relation to the maximum possible depression severity that can be achieved in the measurement tool ^14^.

***Five Top Features:***

***Global functioning***

As described above, GAF ^4^ was evaluated by trained raters to assess participants’ psychological, social, and occupational functioning on a scale from 1 (severely impaired) to 100 (extremely high functioning). In the study population in-& outpatient multisite sample, the split version of the GAF ^18^ was used which divides the scale into disability and symptomatic functioning. The mean of the two values was calculated to harmonize this variable with the single score available in the other sites.

***Childhood maltreatment***

The emotional abuse subscale of the CTQ ^7^ described above was included in the sparse model. No harmonization between sites was necessary.

***Somatic symptoms***

The somatization subscale of the SCL-90-R ^9^ as described above was included in the sparse model. No harmonization between sites was necessary.

***Big Five Personality dimensions***

Neuroticism and extraversion both emerged as features with most predictive value for the sparse model. As Big Five personality dimensions were assessed with different instruments between the sites (NEO-FFI ^10^, BFI-2-S ^11^, BFI-S ^19^), they were transformed into POMP scores as described above, to allow for inter-site comparability ^13,14^.

## Recruitment procedures and site information

*Study population inpatients, site #1*

For the study population inpatients, site #1, data sets from two independent cohorts were combined: the Marburg–Münster-Affective-Disorders-Cohort-Study (MACS - Münster site; ^20^) and the Münster-Neuroimaging-Cohort (MNC; ^21^). From both ongoing studies that comprise participants between 18–65 years of age, those with a confirmed MDD diagnosis during an acute depressive episode according to the German version of the Structured Clinical Interview for DSM-IV (SCID; ^22^), who were currently undergoing inpatient treatment at the Department of Psychiatry, University Hospital Münster during the recruitment interval between 07/2015 and 06/2021 were selected. Any history of neurological (e.g., concussion, stroke, tumor, neuroinflammatory diseases) and medical (e.g., cancer, chronic inflammatory or autoimmune diseases, heart diseases, diabetes mellitus, infections) conditions were exclusion criteria. Patients received inpatient treatment as usual, including psychiatric medication and CBT-based psychotherapy.

*Real-World inpatients, site #1*

The real-world inpatients, site #1 sample consisted of psychiatric inpatients, aged 18 years or older, at the Department of Psychiatry, University Hospital Münster. Criteria for initial eligibility were the admission to any of the open adult inpatient services of the hospital during the recruitment period between 03/2019 and 11/2021, treatment duration of more than three days and the diagnosis of major depressive disorder (MDD) at the time of admission. Patients received inpatient treatment as usual, including psychiatric medication, CBT-based psychotherapy, electro-convulsive therapy (ECT), and repetitive transcranial magnetic stimulation (rTMS).

*Study population in-& outpatients, site #1*

The study population in- & outpatients, site #1 sample comprises participants between 18–65 years of age, those with a confirmed MDD diagnosis during an acute depressive episode according to the German version of the SCID, who were currently undergoing inpatient treatment at the Department of Psychiatry, University Hospital Münster or outpatient treatment at the psychotherapeutic outpatient unit, University Münster between 07/2020 and 09/2023. Any history of neurological (e.g., concussion, stroke, tumor, neuroinflammatory diseases) and medical (e.g., cancer, chronic inflammatory or autoimmune diseases, heart diseases, diabetes mellitus, infections) conditions were exclusion criteria. Patients received inpatient treatment including psychotherapy, medication, and ECT, or outpatient CBT-based psychotherapy and medication and were assessed again at the conclusion of their treatment.

*Study population inpatients, site #2*

For the study population inpatients, site #2 sample, the Marburg site of the MACS cohort study was used. Participant selection and recruitment procedures were identical to study population inpatients at site #1, but all patients were treated and recruited at the Department of Psychiatry and Psychotherapy, University of Marburg during the recruitment interval between 09/2014 and 05/2021. Patients received inpatient treatment as usual, including psychiatric medication and CBT-based psychotherapy.

*Study population inpatients, site #3*

For study population inpatients, site #3, participant selection and recruitment procedures were again harmonized to procedures from study population inpatients at sites #1 and #2, but all patients were treated and recruited at the Department of Psychiatry and Psychotherapy, University Hospital Jena and included for participation between 09/2022 and 12/2023. Patients received inpatient treatment as usual, including psychiatric medication, CBT-based psychotherapy, ECT, and rTMS.

*Study population in- & outpatients, multisite*

For the study population in- & outpatient multisite sample, participants were recruited at ten international sites as part of the Personalized Prognostic Tools for Early Psychosis Management (PRONIA; https://www.pronia.eu/) study, following a standardized recruitment and ascertainment protocol (see ^23^). Participants were recruited between 02/2014 and 11/2017. The recruitment sites included the Department of Psychiatry and Psychotherapy, Ludwig-Maximilians-University Munich, Germany; Department of Psychiatry and Psychotherapy, University of Cologne, Germany; Heinrich Heine University Düsseldorf, Germany; University of Münster, Germany; Department of Pathophysiology and Transplantation, University of Milan, Italy; University of Bari Aldo Moro, Italy;  University of Birmingham, United Kingdom; Department of Psychiatry, University of Udine, Italy; Department of Psychiatry, University of Turku, Finland; Department of Psychiatry and Psychotherapy, University of Basel, Switzerland. General inclusion criteria of the PRONIA study were age between 15 and 40 years, sufficient language skills for participation as well as capacity to provide informed consent/assent. General exclusion criteria were an IQ below 70, current or past head trauma with loss of consciousness (> 5 minutes), current or past known neurological or somatic disorders potentially affecting the structure or functioning of the brain, current or past alcohol dependence, or polysubstance dependence within the past six months, and any medical indication against MRI. Participants were selected for this sample if they met criteria for recent onset depression, meaning a diagnosis of MDD within the past 3 months, as established by SCID. Specific exclusion criteria for this sample were: (1) a previous episode of DSM-IV-TR major depression prior to the current or recent episode, and (2) a duration of the current episode exceeding 24 months. Participants received treatment as usual, including psychiatric medication, inpatient or outpatient psychotherapy, and counselling sessions.

*Real-world  inpatients, site #4*

The real-world inpatients sample #4 comprises participants who were recruited at the Department of Psychiatry and Psychotherapy of the LMU University Hospital, LMU Munich (German Clinical Trial Register ID: DRKS00019821). Patients between the age of 18 to 65 who were admitted to a structured 10-week inpatient psychotherapy program of Cognitive-Behavioral-Analysis System of Psychotherapy (CBASP; ^24^) were eligible to take part in the ongoing naturalistic study. This program is primarily indicated for patients with chronic depression and general CBASP eligibility was assessed in a preliminary medical interview. Additionally, the main diagnosis of a persistent depressive disorder (PDD) was verified with SCID after admission. Patients with acute suicidality, unstable treatable somatic illnesses, such as acute or chronic infections, a current pregnancy or patients admitted to a second or subsequent CBASP booster session, were excluded. Baseline measures took place between 06/2018 and 01/2023, all within the first 10 days of treatment while post measures were taken at the earliest 10 days before the planned discharge after 10 weeks.

*Real-world  outpatients, site #5*

The real-world outpatients, site #5 sample comprises participants with a confirmed MDD diagnosis during an acute depressive episode according SCID, aged 18 years or older, who were currently undergoing outpatient treatment at the psychotherapeutic outpatient unit, University of Halle. Criteria for initial eligibility were the admission of adult outpatient services, a diagnosis of major depressive disorder (MDD) at the time of admission, and an outpatient treatment for at least 6 weeks. All patients received CBT-based psychotherapy and data were assessed between 09/2020 and 01/2024.

*Real-world  outpatients, site #6*

The real-world outpatients, site #6 sample includes participants with a confirmed MDD diagnosis according to SCID, who were currently undergoing CBT-based outpatient treatment at the psychotherapeutic outpatient unit, University Münster. Participants were recruited from 03/2016 to 04/2021. All participants who were additionally recruited to take part in the study population in/ & outpatients, site #1 study during their treatment were excluded from this sample to make sure the samples were independent.

*Real-world general population sample*

For the real-world general population sample, participants from the general population 20-69 years old were invited to the study center, where an extensive program including interview, touch screen and various examinations took place between 03/2016 and 05/2019. For a detailed description see ^25^. From all 10 000 participants at the Halle (Saale) site, those who self-reported having received a formal MDD diagnosis at some point in their lives were selected as the real-world general population sample.

*Note:* While the sample names were chosen to distinguish between samples in which homogenous populations were expected due to selective inclusion criteria (study population) and those who were expected to be more heterogeneous (real-world samples), the data for the real-world samples were nevertheless collected for research purposes as part of naturalistic studies and not extracted from the electronic health record. Some selection effects from patients declining to provide data are therefore to be expected.

# Results

### *Supplementary analyses on differences in features between external validation samples*

To assess potential differences between the external validation samples and the initial training sample, we performed two additional analyses. First, for each external validation sample, we compared the mean of each variable to the mean of the same variable in the initial base sample (study population inpatients, site #1) using t-tests for continuous variables or chi-square tests for categorical variables. In a second analysis, we compared each external validation sample to the mean of all external validation samples combined. Figure S3 illustrates the effect sizes for these analyses, highlighting the samples that significantly deviated from their respective reference values. The results indicate that samples with the greatest deviation correspond to those exhibiting the weakest performance during external validation. For example, the real-world general population sample, which deviated most markedly from the other sites as participants were not undergoing treatment or currently depressed, reported significantly lower depression severity (d = -0.9, CI[-0.98, -0.83]) and was significantly older (d = 0.72, CI[0.65, 0.80]) compared to the mean of all external validation sites. Model performance was weakest in this sample, as stated in the main results (*r*_1227_= 0.48). This is in line with the aim of improving representative sampling for clinical symptom prediction in mental health to aid generalization performance.

### *Supplementary analysis of generalization performance excluding neuroticism*

To determine the extent to which the generalizability of our predictive model relied on neuroticism, a trait strongly correlated with depressive symptoms ^27^, we conducted an additional analysis to explicitly test the generalization performance of our model when excluding neuroticism. To this end, we retrained the machine learning model on the remaining top 4 predictive features on study population #1 and tested its performance on all other sites. As expected, generalization performance across all external datasets decreased from *r*_2673_ = 0.60 to *r*_2673_ = 0.50, but remained significant (*p* < 0.001) when excluding neuroticism. Likewise, standard deviation increased from 0.089 to 0.121. Investigating performance on the nine samples separately excluding neuroticism shows that performance on all sites decreased to *r*_1227_ = 0.26 in the real-world general population sample, remained unchanged in real-world outpatients #6 (*r*_250_ = 0.50), and only slightly decreased to *r*_350_ = 0.69 in real-world inpatients #1 (from *r*_350_ = 0.73). Thus, all but the lowest performances (real-world general population sample) were within 1.41 standard deviations of the mean of the sparse model performance without neuroticism (*r*_364_ = 0.62, *SD* = 0.123).

We repeated this analysis to also evaluate the performance if global functioning (GAF) was excluded, as this is another variable that is typically strongly related to depression severity. For the model without GAF, r = 0.574, with a BESD success rate of 78.71 % was found. When both neuroticism and GAF were excluded, we observed a r = 0.579, corresponding to a BESD success rate of 78.96 %. These results demonstrate that the exclusion of GAF alone, or in combination with neuroticism, results in only a marginal decrease in predictive performance. These supplementary analyses reinforce the robustness of the model, which continues to perform well even without the most highly weighted features of the original model.

**Fig. S1. Feature Permutation Importance from Elastic Net Modeling.**
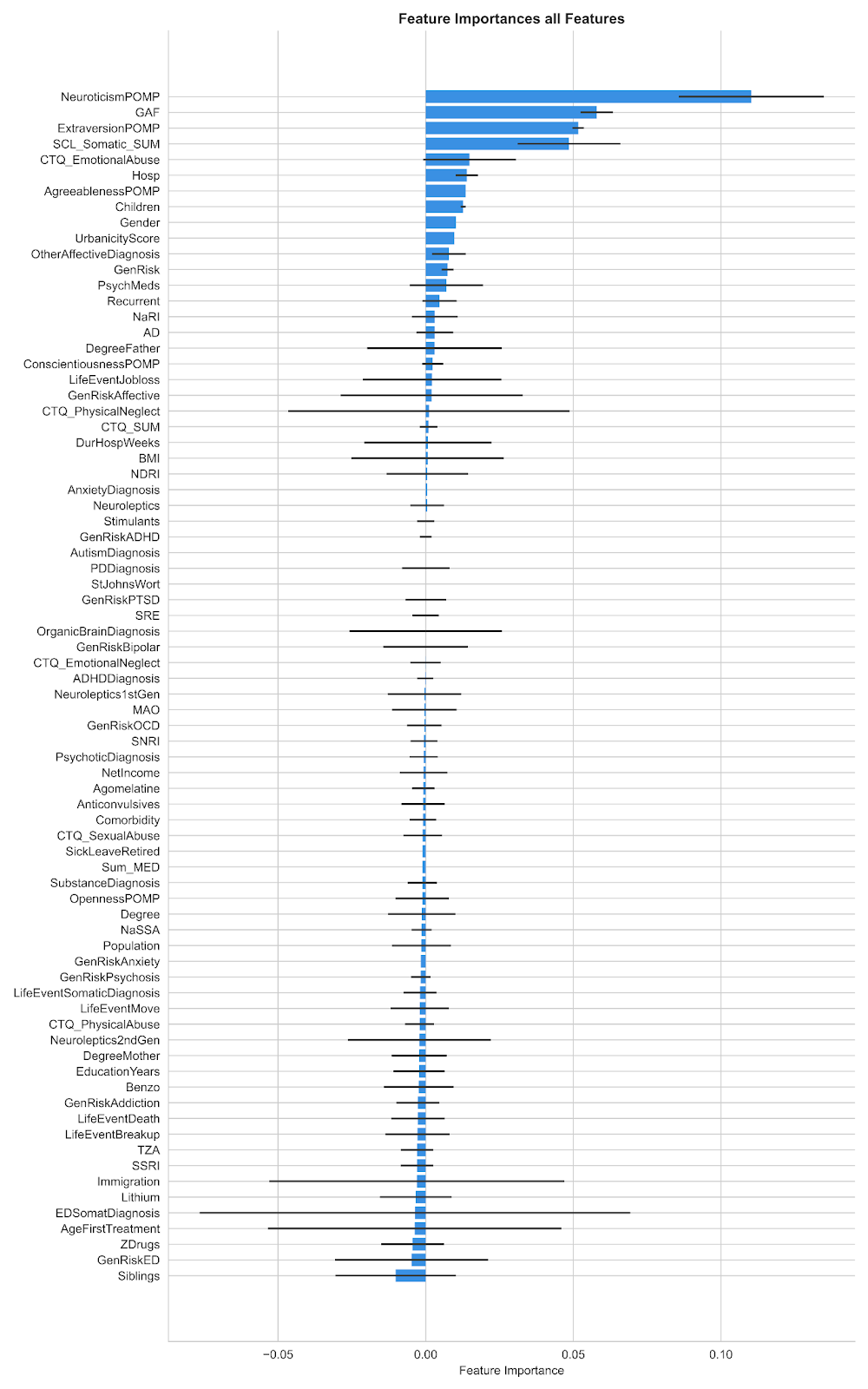


Permutation importance ranking of features is depicted, gauging the influence of each on the model's predictive error when permuted. The vertical bars manifest the average importance scores, and the error bars extending horizontally denote one standard deviation from this mean, capturing the consistency of feature significance. This representation underscores the relative contribution and stability of each feature within the model's architecture.

**Fig. S2. Mean Absolute Error (MAE) Analysis for Base Model Predictions.**


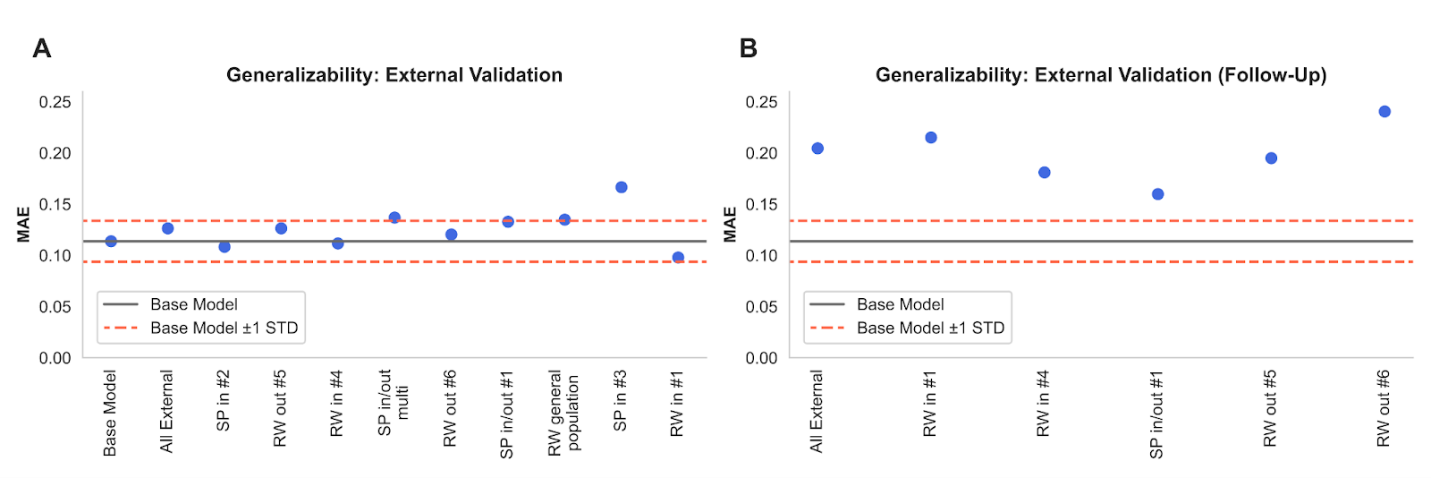


(A) Baseline Depression Severity Predictions: Model performance across nine external validation sites, with a solid line indicating the base model's MAE and dashed lines representing the MAE ±1 STD. Individual data points denote the site-specific MAE. (B) Follow-Up Depression Severity Predictions: Comparable depiction of the model’s predictive accuracy for follow-up depression severity scores.


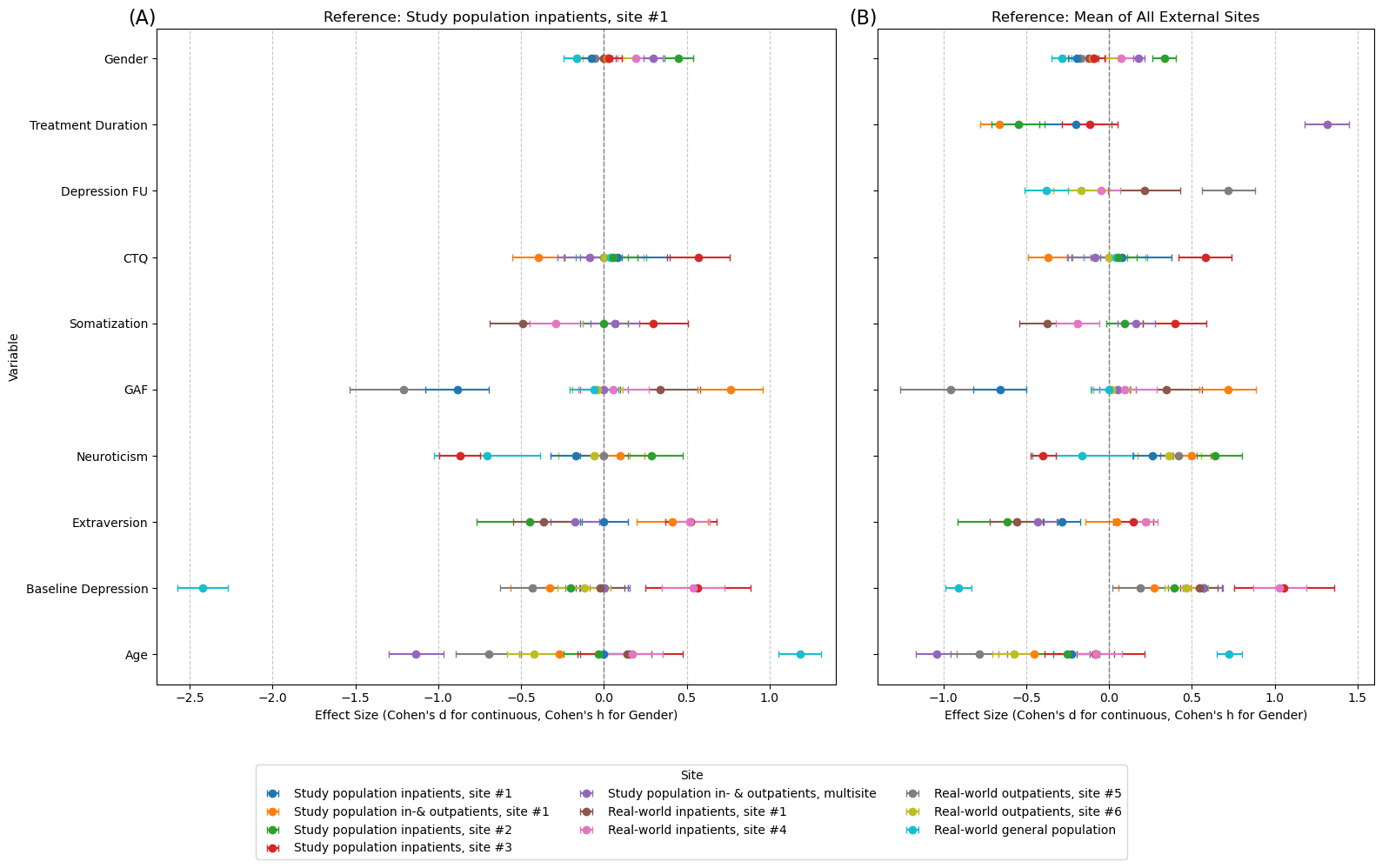
**Fig. S3. Effect size of group differences between external sites in sample characteristics.**

(A) Effect size of group differences between each external site and the initial base sample (study population inpatients, site #1)

(B) Effect size of group differences between each external site and the mean of all external sites

**Table S1.**

Somatic comorbidities that served as exclusion criteria for study population inpatients, site #1.

| **Automatic exclusion from participation** | **Inclusion only on case by case basis, depending on medication status** |
| --- | --- |
| Ankylosing spondylitis with HLA B27 antigen | Allergies |
| Carcinoma | Asthma |
| Colitis Ulcerosa | Borreliosis |
| Crohn’s disease | Chronic bronchitis |
| Encephalopathy | Chronic leukozytosis |
| Epilepsy | Diabetes mellitus type I |
| Hereditary postural tremor | Hemochromatosis |
| Facioscapulohumeral muscular dystrophy | Hashimoto’s thyreoidits |
| Hepatitis C | Hepatitis B |
| Lupus | Lichen sclerosus |
| Multiple Sclerosis | Lymphocytic thyroiditis |
| Narcolepsy | Meningitis |
| Neuroborreliosis | Migraine with aura |
| Neural muscle atrophy | Atopic dermatitis |
| Sarcoidosis | Neurofibromatosis type I |
| Sjögren syndrome | Paroxysmal hemicrania |
| Scleroderma | Proctitis ulcerosa |
| Undifferenciated connective tissue disease | Rheumatic disorders |
| Stroke | Restless legs syndrome |
| Malignant or semi-malignant tumor | Sleep apnea |
| Tumor in the head region (including benign) | Tinnitus |
| Rheumatoid arthritis | Tuberculosis |
|  | Gitelman syndrome |
|  | Psoriasis |
|  | Disorders of antidiuretic hormone (ADH) secretion |

**Table S2.**

Group comparison results between study population inpatients and real-world inpatients from site #1.

| **Dimension** | **Variables** | **Study population inpatients, site #1** | **N** | **Real-world inpatients, site #1** | **N** | **Test statistic** | **Effect size**  **(95% CI)** | **p** | **p_FDR_** |
| --- | --- | --- | --- | --- | --- | --- | --- | --- | --- |
| **Dimension 1: Sociodemographic variables** |  |  |  |  |  |  |  |  |  |
|  | **Age** |  |  |  |  |  |  |  |  |
|  | Mean  (SD) | 37.40  (12.90) | 366 | 39.41  (17.19) | 345 | -1.76 ^b^ | -.133  (-.280, .014) ^d^ | 0.079 | 0.173 |
|  | Range | 18-66 |  | 18-81 |  |  |  |  |  |
|  | **Gender** |  |  |  |  |  |  |  |  |
|  | male/female | 158/208 | 366 | 162/183 | 345 | 1.29 ^b^ | 0.858 (0.638, 1.153) ^e^ | 0.327 | 0.483 |
|  | **Years in formal education** |  |  |  |  |  |  |  |  |
|  | Mean  (SD) | 13.85  (2.56) | 364 | 12.68  (2.78) | 343 | 5.86 ^b^ | 0.441 (0.292, 0.590) ^d^ | **<0.0001** | **<0.0001** |
|  | **Highest degree (self)** |  |  |  |  |  |  |  |  |
|  | Mean rank | 403.96 | 362 | 299.22 | 343 | 43636.50 ^c^ | 0.261 ^f^ | **<0.0001** | **<0.0001** |
|  | **Highest degree (father)** |  |  |  |  |  |  |  |  |
|  | Mean rank | 275.68 | 217 | 280.31 | 339 | 36168.50 ^c^ | 0.014 ^f^ | 0.738 | 0.797 |
|  | **Highest degree (mother)** |  |  |  |  |  |  |  |  |
|  | Mean rank | 270.78 | 219 | 285.13 | 339 | 35211.00 ^c^ | 0.044 ^f^ | .365 | 0.502 |
|  | **Household income** |  |  |  |  |  |  |  |  |
|  | Mean  (SD) | 2552.65  (1898.15) | 186 | 2550.85 (4826.40) | 315 | 0.005 ^b^ | 0.000 (-0.181, 0.182) ^d^ | 0.996 | 0.999 |
|  | **Population birth place** |  |  |  |  |  |  |  |  |
|  | Mean  (SD) | 138971.26  (283312.75) | 152 | 223608.85  (1305434.69) | 336 | -0.791 ^b^ | -0.077 (-0.269, 0.114) ^d^ | 0.430 | 0.562 |
|  | **Urbanicity Score** |  |  |  |  |  |  |  |  |
|  | Mean Rank | 275.02 | 143 | 225.10 | 336 | 19016.00 ^c^ | 0.177 ^f^ | **<0.0001** | **<0.0001** |
|  | **Immigration** |  |  |  |  |  |  |  |  |
|  | yes/no | 27/177 | 204 | 59/284 | 343 | 1.52 ^b^ | 1.36 (0.830, 2.229) ^e^ | 0.227 | 0.368 |
|  | **Children** |  |  |  |  |  |  |  |  |
|  | yes/no | 72/152 | 224 | 133/210 | 343 | 2.58 ^b^ | 1.337 (0.938, 1.096) ^e^ | 0.128 | 0.249 |
|  | **Siblings** |  |  |  |  |  |  |  |  |
|  | yes/no | 202/21 | 223 | 308/35 | 343 | 0.094 ^b^ | 0.915 (0.518, 1.617) ^e^ | 0.886 | 0.913 |
| **Dimension 2: Depressive symptom**  **severity** |  |  |  |  |  |  |  |  |  |
|  | **BDI** |  |  |  |  |  |  |  |  |
|  | Mean  (SD) | 25.08  (10.72) | 366 | 24.76  (11.40) | 345 | 0.389 ^b^ | 0.029 (-0.118, 0.176) ^d^ | 0.698 | 0.780 |
|  | **HAMD** |  |  |  |  |  |  |  |  |
|  | Mean  (SD) | 15.84  (6.50) | 364 | 17.17  (6.51) | 316 | -2.67 ^b^ | -0.205 (-0.356, -0.054) ^d^ | **0.0078** | **0.0286** |
|  | **GAF** |  |  |  |  |  |  |  |  |
|  | Mean  (SD) | 54.43  (9.37) | 364 | 53.78  (9.24) | 327 | 0.919 ^b^ | 0.070 (-0.079, 0.219) ^d^ | 0.358 | 0.502 |
|  | **Recurring depression** |  |  |  |  |  |  |  |  |
|  | yes/no | 270/95 | 365 | 230/115 | 345 | 4.55 ^b^ | 0.794 (0.509, 0.973) ^e^ | **0.0396** | 0.0997 |
|  | **Psychiatric comorbidity** |  |  |  |  |  |  |  |  |
|  | yes/no | 207/159 | 366 | 158/187 | 345 | 8.23 ^b^ | 0.649 (0.483, 0.872) ^e^ | **0.0044** | **0.0175** |
| **Dimension 3: Current psychotropic medication** |  |  |  |  |  |  |  |  |  |
|  | **Medication load** |  |  |  |  |  |  |  |  |
|  | Mean  (SD) | 2.57  (1.79) | 366 | 2.46  (2.22) | 283 | 0.668 ^b^ | 0.054 (-0.101, 0.209) ^e^ | 0.504 | 0.591 |
|  | **Psychotropic medication** |  |  |  |  |  |  |  |  |
|  | yes/no | 332/34 | 366 | 290/55 | 345 | 7.177 ^a^ | 0.540 (0.342, 0.852) ^e^ | **0.0089** | **0.0303** |
|  | **Antidepressant** |  |  |  |  |  |  |  |  |
|  | yes/no | 316/50 | 366 | 258/87 | 345 | 15.246 ^a^ | 0.469 (0.319, 0.689) ^e^ | **<0.0001** | **<0.0001** |
|  | **Agomelatine** |  |  |  |  |  |  |  |  |
|  | yes/no | 37/329 | 366 | 20/325 | 345 | 4.478 ^a^ | 0.547 (0.311, 0.963) ^e^ | **0.0380** | 0.0994 |
|  | **TCA** |  |  |  |  |  |  |  |  |
|  | yes/no | 28/338 | 366 | 21/324 | 345 | 0.676 ^a^ | 0.782 (0.435, 1.406) ^e^ | 0.4603 | 0.580 |
|  | **NRI** |  |  |  |  |  |  |  |  |
|  | yes/no | 2/364 | 366 | 1/344 | 345 | 0.278 ^a^ | 0.529 (0.048, 5.861) ^e^ | 1.0 | 0.999 |
|  | **NDRI** |  |  |  |  |  |  |  |  |
|  | yes/no | 20/346 | 366 | 28/317 | 345 | 1.983 ^a^ | 1.528 (0.844, 2.767) ^e^ | 0.179 | 0.305 |
|  | **SSRI** |  |  |  |  |  |  |  |  |
|  | yes/no | 93/273 | 366 | 96/249 | 345 | 0.531^a^ | 1.132 (0.811, 1.578) ^e^ | 0.497 | 0.591 |
|  | **SNRI** |  |  |  |  |  |  |  |  |
|  | yes/no | 175/191 | 366 | 116/229 | 345 | 14.793 ^a^ | 0.553 (0.408, 0.749) ^e^ | **<0.0001** | **<0.0001** |
|  | **NaSSA** |  |  |  |  |  |  |  |  |
|  | yes/no | 86/280 | 366 | 86/259 | 345 | 0.198 ^a^ | 1.081 (0.767, 1.524) ^e^ | 0.662 | 0.763 |
|  | **MAOI** |  |  |  |  |  |  |  |  |
|  | yes/no | 6/360 | 366 | 6/339 | 345 | 0.011 ^a^ | 1.062 (0.339, 3.325) ^e^ | 1.0 | 0.999 |
|  | **SRE** |  |  |  |  |  |  |  |  |
|  | yes/no | 0/366 | 366 | 2/343 | 345 | 2.13 ^a^ | 1.006 (0.998, 1.014) ^e^ | 0.235 | 0.372 |
|  | **Neuroleptics** |  |  |  |  |  |  |  |  |
|  | yes/no | 151/215 | 366 | 166/179 | 345 | 3.382 ^a^ | 1.320 (0.982, 1.776) ^e^ | 0.0703 | 0.159 |
|  | **1^st^ generation neuroleptics** |  |  |  |  |  |  |  |  |
|  | yes/no | 45/321 | 366 | 50/295 | 345 | 0.741 ^a^ | 1.209 (0.784, 1.864) ^e^ | 0.440 | 0.565 |
|  | **2^nd^ generation neuroleptics** |  |  |  |  |  |  |  |  |
|  | yes/no | 130/236 | 366 | 141/204 | 345 | 2.155 ^a^ | 1.255 (0.927, 1.699) ^e^ | 0.143 | 0.270 |
|  | **Lithium** |  |  |  |  |  |  |  |  |
|  | yes/no | 18/348 | 366 | 9/336 | 345 | 2.593 ^a^ | 0.518 (0.229, 1.169) ^e^ | 0.119 | 0.244 |
|  | **Anticonvulsives** |  |  |  |  |  |  |  |  |
|  | yes/no | 10/356 | 366 | 18/327 | 345 | 2.899 ^a^ | 1.960 (0.892, 4.307) ^e^ | 0.122 | 0.244 |
|  | **Stimulants** |  |  |  |  |  |  |  |  |
|  | yes/no | 0/366 | 366 | 8/338 | 345 | 8.584 ^a^ | 1.024 (1.007, 1.041) ^e^ | **0.0029** | **0.0131** |
|  | **Benzodiazepines** |  |  |  |  |  |  |  |  |
|  | yes/no | 3/363 | 366 | 42/303 | 345 | 38.619 ^a^ | 16.773 (5.148, 54.648) ^e^ | **<0.0001** | **<0.0001** |
|  | **Z-drugs** |  |  |  |  |  |  |  |  |
|  | yes/no | 4/362 | 366 | 25/320 | 345 | 17.188 ^a^ | 7.070 (2.435, 20.533) ^e^ | **<0.0001** | **<0.0001** |
| **Dimension 4: Family and own**  **psychiatric history** |  |  |  |  |  |  |  |  |  |
|  | **Genetic risk total** |  |  |  |  |  |  |  |  |
|  | yes/no | 115/77 | 192 | 173/137 | 310 | 0.811 ^a^ | 0.846 (0.587, 1.218) ^e^ | 0.404 | 0.539 |
|  | **Genetic risk major depressive disorder** |  |  |  |  |  |  |  |  |
|  | yes/no | 72/136 | 208 | 123/220 | 343 | 0.088 ^a^ | 1.056 (0.736, 1.515) ^e^ | 0.783 | 0.819 |
|  | **Genetic risk bipolar disorder** |  |  |  |  |  |  |  |  |
|  | yes/no | 1/198 | 199 | 15/328 | 343 | 6.586 ^a^ | 9.055 (1.187, 69.077) ^e^ | **0.0080** | **0.0286** |
|  | **Genetic risk substance use disorder** |  |  |  |  |  |  |  |  |
|  | yes/no | 29/178 | 207 | 49/294 | 343 | 0.008 ^a^ | 1.023 (0.623, 1.679) ^e^ | 1.00 | 0.999 |
|  | **Genetic risk anxiety disorder** |  |  |  |  |  |  |  |  |
|  | yes/no | 9/190 | 199 | 27/316 | 343 | 2.278 ^a^ | 1.804 (0.831, 3.917) ^e^ | 0.154 | 0.283 |
|  | **Genetic risk obsessive-compulsive disorder** |  |  |  |  |  |  |  |  |
|  | yes/no | 3/196 | 199 | 4/339 | 343 | 0.115 ^a^ | 0.711 (0.171, 3.48) ^e^ | 0.711 | 0.780 |
|  | **Genetic risk posttraumatic stress disorder** |  |  |  |  |  |  |  |  |
|  | yes/no | 1/198 | 199 | 14/329 | 343 | 5.995 ^a^ | 8.426 (1.100, 64.565) ^e^ | **0.0133** | **0.0431** |
|  | **Genetic risk psychosis** |  |  |  |  |  |  |  |  |
|  | yes/no | 9/191 | 200 | 11/332 | 343 | 0.595 ^a^ | 0.703 (0.286, 1.727) ^e^ | 0.482 | 0.591 |
|  | **Genetic risk ADHD** |  |  |  |  |  |  |  |  |
|  | yes/no | 1/198 | 199 | 13/330 | 343 | 5.409 ^a^ | 7.800 (1.013, 60.081) ^e^ | **0.022** | **0.0623** |
|  | **Genetic risk eating disorder** |  |  |  |  |  |  |  |  |
|  | yes/no | 5/193 | 198 | 16/327 | 343 | 1.540 ^a^ | 1.889 (0.681, 5.237) ^e^ | 0.254 | 0.393 |
|  | **Age at first psychiatric treatment** |  |  |  |  |  |  |  |  |
|  | Mean  (SD) | 31.18  (12.27) | 338 | 28.69  (16.04) | 339 | 2.266 ^b^ | 0.174 (0.023, 0.325) ^d^ | **0.024** | **0.0653** |
|  | **Number of hospitalizations** |  |  |  |  |  |  |  |  |
|  | Mean  (SD) | 2.29  (2.02) | 365 | 2.83  (4.68) | 341 | -1.947 ^b^ | -0.150 (-0.298, -0.002) ^d^ | 0.052 | 0.126 |
|  | **Duration of hospitalizations**  **(total in weeks)** |  |  |  |  |  |  |  |  |
|  | Mean  (SD) | 10.34  (17.69) | 363 | 31.25  (33.94) | 236 | -8.724 ^b^ | -0.824 (-0.994, -0.654) ^d^ | **<0.0001** | **<0.0001** |
|  | **Duration on sick leave/retired (due to mental illness)**  **(total in months)** |  |  |  |  |  |  |  |  |
|  | Mean  (SD) | 10.39  (31.48) | 330 | 19.04  (45.62) | 340 | -2.865 ^b^ | -0.220 (-0.372, -0.068) ^d^ | **0.0043** | **0.0175** |
| **Dimension 5: Childhood maltreatment and stressful life events** |  |  |  |  |  |  |  |  |  |
|  | **CTQ Sum** |  |  |  |  |  |  |  |  |
|  | Mean  (SD) | 46.30  (16.18) | 358 | 47.17  (16.94) | 338 | -0.693 ^b^ | -0.053 (-0.201, 0.096) ^d^ | 0.488 | 0.591 |
|  | **CTQ physical neglect** |  |  |  |  |  |  |  |  |
|  | Mean  (SD) | 8.11  (3.03) | 361 | 11.55  (5.82) | 342 | -9.80 ^b^ | -0.751 (-0.904, -0.598) ^d^ | **<0.0001** | **<0.0001** |
|  | **CTQ emotional neglect** |  |  |  |  |  |  |  |  |
|  | Mean  (SD) | 13.87  (5.60) | 362 | 13.74  (5.76) | 341 | 0.306 ^b^ | 0.023 (-0.125, 0.171) ^d^ | 0.760 | 0.808 |
|  | **CTQ physical abuse** |  |  |  |  |  |  |  |  |
|  | Mean  (SD) | 7.03  (3.52) | 361 | 7.13  (3.69) | 342 | -0.371 ^b^ | -0.028 (-0.176, 0.120) ^d^ | 0.711 | 0.780 |
|  | **CTQ emotional abuse** |  |  |  |  |  |  |  |  |
|  | Mean  (SD) | 11.18  (5.49) | 360 | 11.56  (5.82) | 342 | -0.897 ^b^ | -0.068 (-0.216, 0.080) ^d^ | 0.369 | 0.502 |
|  | **CTQ sexual abuse** |  |  |  |  |  |  |  |  |
|  | Mean  (SD) | 6.23  (3.33) | 361 | 6.70  (3.78) | 341 | -1.737 ^b^ | -0.132 (-0.280, 0.017) ^d^ | 0.083 | 0.176 |
|  | **Separation or divorce** |  |  |  |  |  |  |  |  |
|  | yes/no | 36/116 | 152 | 62/280 | 342 | 2.042 ^a^ | 0.713 (0.449, 1.135) | 0.179 | 0.305 |
|  | **Death of a loved one** |  |  |  |  |  |  |  |  |
|  | yes/no | 20/132 | 152 | 57/285 | 342 | 0.985 ^a^ | 1.320 (0.762, 2.287) | 0.350 | 0.502 |
|  | **Job loss** |  |  |  |  |  |  |  |  |
|  | yes/no | 23/129 | 152 | 37/305 | 342 | 1.834 ^a^ | 0.680 (0.389, 1.191) | 0.182 | 0.305 |
|  | **Moved house** |  |  |  |  |  |  |  |  |
|  | yes/no | 31/121 | 152 | 70/272 | 342 | 0.000 ^a^ | 1.005 (0.625, 1.614) | 1.0 | 0.999 |
| **Dimension 6: Somatic symptoms** |  |  |  |  |  |  |  |  |  |
|  | **SCL-90-R somatization subscale** |  |  |  |  |  |  |  |  |
|  | Mean  (SD) | 10.91  (8.45) | 196 | 12.29  (7.81) | 343 | -1.918 ^b^ | -0.172 (-0.347, 0.004) ^d^ | 0.056 | 0.131 |
|  | **BMI** |  |  |  |  |  |  |  |  |
|  | Mean  (SD) | 25.91  (5.34) | 340 | 27.34  (8.93) | 44 | -1.039 ^b^ | -0.244 (-0.559, 0.070) ^d^ | 0.304 | 0.459 |
| **Dimension 7: Big Five Personality Dimensions** |  |  |  |  |  |  |  |  |  |
|  | **Neuroticism** |  |  |  |  |  |  |  |  |
|  | Mean  (SD) | 66.95  (15.50) | 350 | 68.61  (17.34) | 336 | -1.326 ^b^ | -0.101 (-0.251, 0.048) ^d^ | 0.184 | 0.305 |
|  | **Extraversion** |  |  |  |  |  |  |  |  |
|  | Mean  (SD) | 39.08  (14.53) | 351 | 36.12  (18.71) | 335 | 2.307 ^b^ | 0.177 (0.027, 0.327) ^d^ | **0.021** | 0.0621 |
|  | **Openness** |  |  |  |  |  |  |  |  |
|  | Mean  (SD) | 58.15  (14.06) | 351 | 54.91  (20.73) | 335 | 2.385 ^b^ | 0.184 (0.033, 0.333) ^d^ | **0.017** | 0.0525 |
|  | **Agreeableness** |  |  |  |  |  |  |  |  |
|  | Mean  (SD) | 64.62  (13.13) | 350 | 68.32  (15.59) | 337 | -3.360 ^b^ | -0.257 (-0.408, -0.107)^d^ | **0.00083** | **0.00403** |
|  | **Conscientiousness** |  |  |  |  |  |  |  |  |
|  | Mean  (SD) | 60.37  (15.81) | 350 | 55.65  (19.23) | 337 | 3.505 ^b^ | 0.269 (0.118, 0.419) ^d^ | **0.00049** | **0.0025** |

*Abbreviations.* ADHD = Attention Deficit Hyperactivity Disorder, BDI = Beck Depression Inventory, BMI = Body Mass Index, CI = Confidence Interval, CTQ = Childhood Maltreatment Questionnaire, GAF = General Assessment of Functioning, HAMD = Hamilton Depression Rating Scale, MAOI = Monoamine Oxidase Inhibitors, NaSSA = Noradrenergic and Specific Serotonergic Antidepressants, NDRI = Norepinephrine–Dopamine Reuptake Inhibitor , NRI = Norepinephrine Reuptake Inhibitor, SCL = Symptom Checklist, SNRI = Serotonin–Norepinephrine Reuptake Inhibitor, SRE = Serotonin Reuptake Enhancer, SSRI = Selective Serotonin Reuptake Inhibitor, TCA = Tricyclic Antidepressant.

*Note.* Analysis method and effect size estimate varied depending on assumptions of the tests and is indicated as follows: ^a^ X²-test, ^b^ two-sample t-test, ^c^ Mann-Whitney U test, ^d^ cohen’s d, ^e^ odd’s ratio, ^f^ pearson’s r.

**Table S3. Results for classification of severely depressed non-responders.**

| **Site** | **N_train** | **N_test** | **balanced accuracy** |
| --- | --- | --- | --- |
| Study population inpatients, site #2 | 723 | 67 | 0.860656 |
| Real-world inpatients, site #4 | 662 | 128 | 0.713705 |
| Study population in-& outpatients, site #1 | 732 | 58 | 0.680000 |
| Study population inpatients, site #1 | 635 | 155 | 0.652864 |
| Real-world inpatients, site #1 | 617 | 173 | 0.634588 |
| Real-world outpatients, site #5 | 708 | 82 | 0.580128 |
| Real-world outpatients, site #6 | 663 | 127 | 0.500000 |

**References**

1. Lederbogen F, Kirsch P, Haddad L, et al. City living and urban upbringing affect neural social stress processing in humans. *Nature*. 2011;474(7352):498-501. doi:10.1038/nature10190

2. Beck AT, Steer RA, Brown GK. Beck depression inventory. *San Antonio, TX*. Published online 1987.

3. Hamilton M. Development of a Rating Scale for Primary Depressive Illness. *British Journal of Social and Clinical Psychology*. 1967;6(4):278-296.

4. Hall RCW. Global Assessment of Functioning: A Modified Scale. *Psychosomatics*. 1995;36(3):267-275. doi:10.1016/S0033-3182(95)71666-8

5. Redlich R, Almeida JR, Grotegerd D, et al. Brain morphometric biomarkers distinguishing unipolar and bipolar depression: a voxel-based morphometry–pattern classification approach. *JAMA Psychiatry*. 2014;71(11):1222-1230.

6. Prescribers’ Digital Reference. Published 2022. Accessed September 9, 2022. https://www.pdr.net/

7. Bernstein DP, Fink L, Handelsman L, Foote J. Childhood Trauma Questionnaire. *Assessment of family violence: A handbook for researchers and practitioners*. Published online 1998. doi:https://doi.org/10.1037/t02080-000

8. Norbeck J. Modification of Life Event Questionnaires for Use with Female Respondents. *Res Nurs Health*. 1984;7(1):61-71.

9. Derogatis LR, Savitz KL. The SCL-90-R, Brief Symptom Inventory, and Matching Clinical Rating Scales. Published online 1999. doi:https://doi.org/10.1037/t02080-000

10. Körner A, Drapeau M, Albani C, Geyer M, Schmutzer G, Brähler E. *Deutsche Normierung Des NEO-Fünf-Faktoren-Inventars (NEO-FFI) German Norms for the NEO-Five Factor Inventory*. Vol 17.; 2008.

11. Rammstedt B, Danner D, Soto CJ, John OP. Validation of the Short and Extra-Short Forms of the Big Five Inventory-2 (BFI-2) and Their German Adaptations. *European Journal of Psychological Assessment*. 2020;36(1):149-161. doi:10.1027/1015-5759/a000481

12. Soto CJ, John OP. The next Big Five Inventory (BFI-2): Developing and assessing a hierarchical model with 15 facets to enhance bandwidth, fidelity, and predictive power. *J Pers Soc Psychol*. 2017;113(1):117.

13. Fleeson W, Gallagher P. The Implications of Big Five Standing for the Distribution of Trait Manifestation in Behavior: Fifteen Experience-Sampling Studies and a Meta-Analysis. *J Pers Soc Psychol*. 2009;97(6):1097-1114. doi:10.1037/a0016786

14. Cohen P, Cohen J, Aiken LS, West SG. The problem of units and the circumstance for POMP. *Multivariate Behav Res*. 1999;34(3):315-346.

15. Dustin Wood. fancyr: Fancy Statistics for Correlational (r) Analyses. Published online 2023.

16. Beck AT, Steer RA, Brown GK. *Beck Depression Inventory (BDI-II)*. Pearson; 1996.

17. Kroenke K, Spitzer RL. The PHQ-9: a new depression diagnostic and severity measure. *Psychiatr Ann*. 2002;32(9):509-515.

18. Karterud S, Pedersen G, Loevdahl H, Friis S. Global Assessment of Functioning--Split Version (S-GAF): Background and Scoring Manual. *Oslo, Norway: Ullevaal University Hospital, Department of Psychiatry*. Published online 1998.

19. Schupp J, Gerlitz JY. BFI-S: Big Five Inventory-SOEP. In: *Zusammenstellung Sozialwissenschaftlicher Skalen. ZIS Version*. Vol 12. A. Glöckner-Rist ; 2008.

20. Kircher T, Wöhr M, Nenadic I, et al. Neurobiology of the major psychoses: a translational perspective on brain structure and function—the FOR2107 consortium. *Eur Arch Psychiatry Clin Neurosci*. 2019;269(8):949-962. doi:10.1007/s00406-018-0943-x

21. Opel N, Redlich R, Dohm K, et al. Mediation of the influence of childhood maltreatment on depression relapse by cortical structure: a 2-year longitudinal observational study. *Lancet Psychiatry*. 2019;6(4):318-326. doi:10.1016/S2215-0366(19)30044-6

22. Wittchen HU, Wunderlich U, Gruschwitz S, Zaudig M. SKID-I: Strukturiertes Klinisches Interview für DSM-IV, Achse I: Psychische Störungen. Published online 1997.

23. Koutsouleris N, Kambeitz-Ilankovic L, Ruhrmann S, et al. Prediction Models of Functional Outcomes for Individuals in the Clinical High-Risk State for Psychosis or With Recent-Onset Depression: A Multimodal, Multisite Machine Learning Analysis. *JAMA Psychiatry*. 2018;75(11):1156-1172. doi:10.1001/jamapsychiatry.2018.2165

24. McCullough Jr JP. Treatment for chronic depression using cognitive behavioral analysis system of psychotherapy (CBASP). *J Clin Psychol*. 2003;59(8):833-846.

25. Peters A, Greiser KH, Göttlicher S, et al. Framework and baseline examination of the German National Cohort (NAKO). *Eur J Epidemiol*. 2022;37(10):1107-1124. doi:10.1007/s10654-022-00890-5

26. Batista GE, Prati RC, Monard MC. A study of the behavior of several methods for balancing machine learning training data. *ACM SIGKDD explorations newsletter*. 2004;6(1):20-29.

27. Navrady LB, Ritchie SJ, Chan SWY, et al. Intelligence and neuroticism in relation to depression and psychological distress: Evidence from two large population cohorts. *European Psychiatry*. 2017;43:58-65. doi:10.1016/j.eurpsy.2016.12.012
